# Editorial: The integrated synapse: the (dys)functional role of neurovascular unit, resident glia and extracellular matrix during synaptic development and plasticity

**DOI:** 10.3389/fncel.2023.1190804

**Published:** 2023-05-17

**Authors:** Ciro De Luca, Nicola Maggio

**Affiliations:** ^1^Laboratory of Neuronal Networks Morphology and Systems Biology, Department of Mental and Physical Health and Preventive Medicine, University of Campania “Luigi Vanvitelli”, Naples, Italy; ^2^Department of Neurology and Neurosurgery, Sackler Faculty of Medicine, Sagol School of Neuroscience, Tel Aviv University, Tel Aviv, Israel; ^3^Department of Neurology, The Chaim Sheba Medical Center at Tel HaShomer, Ramat Gan, Israel

**Keywords:** neurovascular unit (NVU), synaptic plasticity, glymphatic circulation, thrombin (F2), PAR1

The nervous system's intrinsic cellular and extracellular milieu are the keys to unraveling its complexity. However, a specific focus on the vascular system is virtually limited to ischemic, hemorrhagic, and inflammatory diseases. The gateway allowing communication between the central nervous system (CNS) and the body is considered highly selective and non-permissive, creating a barrier, namely the blood-brain barrier (BBB) or blood-spinal cord barrier (BSCB). According to this principle, the barrier should stand while neurons and non-neuronal cells together with the extracellular matrix (ECM) are nurtured by selected metabolites supplied by the general circulation. Moreover, the CNS presents its own fully developed innate surveillance, the microglia without a proper lymphatic system. These classical ideas are being gradually replaced by a progressive understanding of the neurovascular unit and the interactions with the peripheral immune cells (De Luca et al., 2020).

The pleiotropy of coagulation signaling and the existence of the glymphatic during sleep/wake cycles are novel and emergent fields of interest to understand the CNS without cutting out the arteriovenous and cerebrospinal circulation (Akbalut et al., 2023). The neural network is harmonically interplaying with non-neuronal cells to generate electromagnetic fields, using the functional scaffold of ECM for paracrine and autocrine signaling, while the neurovascular unit in both physiology and pathology carries out the metabolic, hormonal, electrolytic- and waste-clearing functions (Virtuoso et al., 2021).

Shavit-Stein et al. demonstrated the role of thrombin and protease-activated receptor 1 (PAR1) in neurotransmission using a model of status epilepticus (SE). SE can impair an important emergent property of the CNS as memory formation fails in proximity to an epileptic seizure; moreover, the SE is coupled by a measurable reduction in long-term potentiation (LTP) in the hippocampal region. Thrombin, the final protease of both the so-called intrinsic and extrinsic coagulation pathways can activate PAR1 (De Luca et al., 2017), and this particular interaction mediates cognitive impairment. The effects of both thrombin and PAR1 inhibition restored neuronal transmission and viability and reduced the transcription of tumor necrosis factor α (TNF-α) (Shavit-Stein et al.).

The SE was also used to study the alterations of brain metabolism influencing long-term plasticity leading to epilepsy. In particular, the organic isothiocyanate compound sulforaphane (SFN) modulating cerebral blood flow demonstrated neuroprotective and anti-inflammatory properties through the metabolic Nrf2 pathway (Daněk et al.). SFN upregulated the Nrf2 pathway and lowered glucose uptake during the acute phase of SE, reversing metabolic impairment in the long term. However, changes in cerebral blood flow and metabolism did not interfere with cell death, rather stimulating the intrinsic resiliency of the CNS to adapt to the epileptogenic insult.

The most investigated cell type to evaluate inflammation and tissue repair is microglia as the intrinsic non-ectodermal immune cell of the CNS (Li and Barres, 2018). Microglia in homeostatic conditions have physiological roles in synaptic plasticity, beyond scavenging, inflammatory, or anti-inflammatory activities. Furthermore, the balance of microglial function is essential for the homeostasis of synaptic transmission and the reshaping of the environment.

The details of how the phenotype of microglia varies according to the environment are not fully elucidated. Membrane proteins, as the translocation protein 18 kDa (TSPO), PAR1-4, receptors for advanced glycation end products (RAGEs), and triggering receptor expressed specifically on myeloid cells 2 (TREM2) coupled with DNAX-activating protein of 12 kDa (DAP12), seem to be crucial as Zhao et al. systematically reviewed.

Micro- and macroglia during neurodegeneration, however, are not able to maintain physiological plasticity, and glial maladaptive changes can be observed before the damage is clinically evident. Sleep abnormalities are among the most investigated prodromal features of Alzheimer's disease (AD) and other neurodegenerative diseases in which microglia and astrocytes are pivotal players (Sunkaria and Bhardwaj, 2022).

Xiao et al. proposed possible mechanisms synergically leading to sleep disturbances and AD, involving an astrocytic imbalance of adenosine receptors (A1-3), dopamine and serotonin regulations, and microglial purinergic and chemokines receptors (namely P2Y12 and CXCR1). The progressive imbalance of these key players results in a pathological phenotype of astrocytes and the release of pro-inflammatory cytokines. In the clinical stages of AD, amyloid-β and tau pathology are also evident. Microglia and astrocytes differently interact with those proteins through apolipoprotein E (ApoE) and TREM2, with failed attempts to resolve the proteinopathy and increased damage to the neurovascular unit.

Neurodegeneration is associated with cognitive impairment and behavioral disturbances, but even psychiatric diseases without relevant neuronal degeneration are characterized by altered synaptic plasticity with glial involvement. Farzinpour et al. focused on the role of microglia/neuron interaction in anxiety-like behaviors in pain. The microglial spine engulfment in the ventral zona incerta was significantly increased in an acute pain model and produced in mice an anxiety-like behavior. Reduced activation of the GABAergic neurons in the zona incerta was measured in these animals; moreover, impairing these neurons through chemogenetic inhibition led to similar results. Furthermore, the pain-sensitization and anxiety-like behavior were reverted, blocking microglial engulfment of neuronal spines through minocycline injection. The capacity of microglia in reshaping synapses according to pathological stimuli is important in determining adaptive or maladaptive plasticity and behavior.

Although non-neuronal cells are increasingly gaining attention for their role in plasticity, neurons should not be conversely underestimated, particularly the role of neural stem cells (NSCs) in health and diseases (Zhang et al.). The regulation and maintenance of NSCs are still puzzling; surprisingly, the Notch signaling that is involved in stemness could regulate a microRNA, namely miR-582-5p, targeting the neuropeptide FAM19A1 as demonstrated both *in vivo* and *in vitro*. Peculiarly, FAM19A1-deficient mice showed altered behavior with hyperactivity, long-term memory impairment, and failure of fear acquisition (Yong et al., 2020). The epigenetic regulation of NSCs is extremely important to promote physiological neurogenesis *in vivo* and to oppose both aging and neurodegeneration.

The role of cellular and molecular components widens the understanding of neurological diseases and leads to the acquisition of general models of neurodegeneration. Neurotransmission is fundamental; however, synaptic plasticity is influenced by a more comprehensive model of systems biology. The present Research Topic collected the latest developments in understanding the role played by each cellular and non-cellular component of the nervous system, with a particular focus on cerebral blood flow, thrombin/plasmin axis, PAR1, and inflammatory response that have shown promising results, altering CNS plasticity.

## Author contributions

CDL and NM conceived of the presented idea. All authors have contributed equally to writing the editorial, read, and approved the final manuscript.
